# Bioavailability of a Novel Form of Microencapsulated Bovine Lactoferrin and Its Effect on Inflammatory Markers and the Gut Microbiome: A Pilot Study

**DOI:** 10.3390/nu10081115

**Published:** 2018-08-17

**Authors:** Clare Dix, Olivia Wright

**Affiliations:** 1 School of Human Movement and Nutrition Science, University of Queensland, Brisbane, QLD 4072, Australia; 2 School of Human Movement and Nutrition Science, University of Queensland, Brisbane, QLD 4072, Australia; o.wright@uq.edu.au

**Keywords:** lactoferrin, immunity, supplement, microbiome, randomised cross-over trial

## Abstract

Bovine lactoferrin, extracted from milk or whey, is used in a range of products to enhance immunity and support digestive health, iron absorption, and homeostasis. This study examined the absorption and effect of Progel (Brisbane, Queensland, Australia) microencapsulated bovine lactoferrin (Inferrin^TM^, Bega Bionutrients, Victoria, Australia) on immune markers and the microbiome. A double-blind randomised, cross-over trial was conducted with 12 healthy males randomised to one of two doses, equivalent to 200 mg or 600 mg lactoferrin, for two four-week supplementation arms, with a two-week washout period. Subjects received either standard bovine lactoferrin or Inferrin^TM^ for each arm. Baseline and post each trial arm, CD69+ activation on CD4+ and CD8+ cells was analysed, bovine and human lactoferrin contents of faecal and serum samples were reported, and the gut microbiome was analysed using 16S sequencing and metagenomic sequencing. The mean level of CD69+ activation on the CD4+ cells was lower after supplementation regardless of the form or dose of lactoferrin. This was statistically significant for the 200 mg dose. A higher level of bovine lactoferrin was found post-supplementation in those taking Inferrin^TM^, although this was not statistically significant. Changes in phylum-level microbial community profiling were detected post-supplementation in the second trial arm, particularly in those receiving Inferrin^TM^. Metagenomic sequencing showed changes in the volumes of the top 100 species of bacteria present before and after all treatment arms. Results suggest that lactoferrin supplementation may have beneficial effects on the microbiome and immune system, and that the use of Inferrin^TM^ improves absorption. Larger detailed studies are needed to ascertain the potential positive effects of bovine lactoferrin supplementation.

## 1. Introduction

Lactoferrin (LF) is an essential glycoprotein involved in a range of functions, including protection against microbial infections [1,2,3] and the modulation of systemic inflammation [4,5,6]. It is found in milk, saliva, tears, and nasal secretions [7]. LF is also expressed by neutrophil leukocytes and microglial cells, both of which express LF in response to inflammation [7]. Bovine lactoferrin (bLF) can be extracted from milk or whey by ion exchange chromatography and is used in a range of infant formula products where it is added to bring levels closer to what is found in human breast milk. It is also becoming a popular supplement to enhance immunity and support digestive health, iron absorption, and homeostasis [8]. However, orally consumed LF may be lost through digestion in the stomach of humans [9]. As such, a method to assist LF in reaching the small intestine would be beneficial. Inferrin^TM^ (Inferrin^TM^, Bega Bionutrients, Victoria, Australia) is a commercially available form of LF. Inferrin^TM^ contains 50% (*w*/*w*) LF microencapsulated using Progel technology. Progel technology was invented by the University of Queensland (Brisbane, Queensland, Australia). The Progel used in Inferrin^TM^ protects LF in low-pH conditions, but releases it in neutral pH environments. This feature enhances the gastro-protection of LF and potentially improves the functionality of LF [10]. The development and properties of microencapsulated LF using Progel technology are described elsewhere [10,11,12,13]. Progel technology has been previously trialled in humans in the form of an encapsulated fish oil in milk (see ACTRN12612000634875).

There are no studies completed using Inferrin^TM^; however, a number of studies have been conducted investigating the potential effects of bLF. Cell culture studies have shown a potential role for bLF in inhibiting infection with influenza type 1 and 2 [14], reducing reactive oxygen species production [15], improving antimicrobial capacity against a range of microbes including *Escherichia coli* and *Staphylococcus aureus* [16], and causing immunosuppression of dendritic cells [17]. Animal models also show positive effects of bLF on immune function, including increased immunoglobin A and immunoglobin M production in small intestine epithelial cells of mice [18], improved natural killer cell production and activity in piglets fed bLF-supplemented formula [19], decreased expression of tumour necrosis factor alpha in healthy mice [20], and decreased bacteria load, neutrophils, and pro-inflammatory cytokines in mice with acute and chronic lung infections [21].

Several human supplementation studies have shown benefits of bLF supplementation for very low birth weight neonates, including reduced incidence of respiratory tract illness, better weight gain, increase haematocrit levels [22], and reduced incidence of sepsis [23,24]. A potential link between changes in the microbiome and occurrence of infectious disease has also been suggested, as bLF supplementation reduced the faecal composition of staphylococci to low levels, which was associated with lower serum levels and reduced infections [25]. In adults, positive effects on immunity were shown in a trial of up to 200 mg bLF per day in a small study of healthy males (*n* = 8), as indicated by statistically significant improvements in total T-cell activation (CD3+, CD4+ and CD8+ cells) [26]. Cell culture of human crypt intestinal epithelial cells treated with human lactoferrin (hLF), bLF, or commercially available bLF, showed modified immune responses with a general trend toward anti-pathogenic activity [27]. More recently, LF supplementation has also been shown to be beneficial in supporting gut health in people taking antibiotics [28].

This study seeks to investigate the absorption and efficacy of Inferrin^TM^, compared to standard bLF without Progel microencapsulation. This could lead to the development of novel functional foods and supplements harnessing the benefits of bLF. The study will also test the efficacy of the bLF and whether it can influence markers of inflammation and infection in vivo. Results will be compared between Inferrin^TM^ and standard forms of bLF. The aims of this project were to: (1) Evaluate the effect of the Inferrin^TM^ on bLF absorption, in vivo; and (2) Evaluate the effect of bLF versus Inferrin^TM^ on immune function on the basis of standard immune system biomarkers. We hypothesised that the increased resistance to gastric degradation of bLF in Inferrin^TM^ and extent of absorption would be higher than that of bLF without Progel microencapsulation as measured by serum and faecal lactoferrin levels.

## 2. Materials and Methods

### 2.1. Ethics Approval

All subjects gave their informed consent for inclusion before they participated in the study. The study was conducted in accordance with the Declaration of Helsinki, and the protocol was approved by the Bellberry Human Research Ethics committee (Approval no. 2015-11-747-FR-1).

### 2.2. Study Population

Twelve participants were recruited from the University of Queensland, Brisbane, Australia through advertisements via the University of Queensland News Newsletter and email lists. These included students and/or staff or relatives of staff of the University of Queensland. The inclusion/exclusion criteria were as follows.

Inclusion criteria: age 18–65 years; male; healthy body weight; available to participate for the required timeframe of the study (10 weeks); not anaemic (i.e., haemoglobin ≥150 ± 20 g/L; haematocrit ≥0.45 ± 0.05; red blood cell (RBC) count ≥5.0 ± 0.5 × 10^12^/L); and non-smoking status.

Exclusion criteria: any allergy or intolerance to dairy products; current consumption of LF-fortified products or LF supplements; inability to provide informed consent due to diminished understanding or comprehension, or a language other than English spoken and an interpreter unavailable; consumption of any form of recreational drug; use of immunosuppressives or any other immunomodulating drugs or analogues; current viral infection (without reference to serology); consumption of >2 standard drinks on any day; or positive smoking status, including those in the process of quitting (i.e., using e-cigarettes and nicotine patches).

### 2.3. Study Design

A double-blind randomised, cross-over trial was conducted with 12 healthy male volunteers at the University of Queensland, School of Human Movement and Nutrition Sciences. Each arm of the trial was four weeks, with a two-week washout period in between. Two doses of bLF were tested (200 mg and 600 mg), with and without Progel microencapsulation (Inferrin^TM^). All participants had fasting blood tests taken at baseline, in the middle, and at the end of each component of the trial period. The total trial period was 10 weeks.

### 2.4. Study Intervention

Participants were randomly allocated to either the 200 mg/day (1 capsule) or 600 mg/day (3 capsules) dose using a block randomisation design by an external party. Participants were then randomised to receive either gelatin capsules containing bLF (Product A) or gelatin capsules containing Inferrin^TM^ (Product B) for the first arm of the trial. They were then given the other product for the second arm of the trial using the same dose (Table 1). Participants consumed supplements once a day in the morning following breakfast.

Capsules containing equivalent doses of LF (Tatura-Bio^TM^ LF from bovine milk; 15% iron saturation level) and Inferrin^TM^ used in this study were provided by Bega Bionutrients (Victoria, Australia).

### 2.5. Outcome Measures

At the start of the trial a full medical history was collected. Demographic and anthropometric (height, weight) data as well as vital signs were also collected. A diet history and 24-h recall were conducted on the first day of the trial using standard protocols (Wollongong Diet History Questionnaire) to check for LF consumption prior to provision of Product A or B. Twenty-four hour recalls were repeated at the end and during each trial arm. Participants fasted for eight hours prior to each trial arm appointment (baseline, two, four weeks) to facilitate collection of fasting blood tests. Faecal samples were collected at the baseline and end of each trial period.

#### 2.5.1. Lactoferrin Absorption

Faecal and serum samples were analysed at baseline and the end of each trial period (i.e., four time points of collection) to measure hLF and bLF levels. Serum LF was measured using a hLF ELISA kit (Cat.No. ab108882, Abcam, Cambridge, UK) and a bLF ELISA kit (Cat.No. KT-668, Kamiya Biomedical Company). Faecal LF levels were measured using Lactoferrin Scan™ (Cat.No. 30351, Techlab, Blacksburg, VA, USA) and a bLF ELISA kit (Cat.No. KT-668, Kamiya Biomedical Company).

#### 2.5.2. CD4+/CD8+/CD69+ Analysis

Analysis of peripheral blood lymphocyte counts, including the expression of CD69+ (a marker of inflammation/immune activation) on CD4+ (T-helper cells) and CD8+ (cytotoxic T cells) was conducted using flow cytometry at the Queensland Brain Institute, University of Queensland, Australia. Percent activation of CD69+ on CD4+ and CD8+ cells was assessed before and after supplementation with LF (Products A and B). The protocol for analysis is outlined elsewhere [26].

#### 2.5.3. Gut Microbiome

Sampling for the gut microbiome was collected by study participants using faecal collection containers and swabs at the same four time points outlined in Section 2.5.1. The gut microbiome was analysed at the Australian Centre for Ecogenomics and Advanced Water Management, University of Queensland, Australia, using 16S sequencing and metagenomic sequencing. Staff from the centre assisted with analysis and interpretation. Frozen faecal samples and serum samples were analysed for both bLF and hLF content pre- and post-supplementation.

An extension of the 16S microbiome analysis was completed to provide more definitive findings. The metagenomics sequencing procedure gives the truest reporting of all of the organisms in the sample without bias, as it avoids the polymerase chain reaction amplification in library construction. Analysing the samples through this system provides a more comprehensive metadata collection which can help pinpoint the changes generated and attributable specifically to the diet changes made, i.e., the LF supplement. All samples were analysed to examine differences between Product A and Product B and the different doses used (200 mg and 600 mg).

### 2.6. Statistical Analysis

Statistical analysis was completed using Statistical Package for the Social Sciences (SPSS) version 24 (IBM Corp. Released 2015. IBM SPSS Statistics for Macintosh, Version 24.0. Armonk, NY, USA: IBM Corp). Continuous and normally distributed variables; age, weight, height, were summarized as means with 95% confidence intervals. Outcome variables were compared between Product A and B groups at before and after each arm using a paired samples *t*-test with normally distributed data, and a related samples Wilcoxon signed rank test if variables were not normally distributed. Statistical significance was assessed as achieving a *p*-value < 0.05.

## 3. Results

All participants tolerated the supplements well. No participant commenced use of other LF supplements or changed their dietary intake of LF throughout the study. There were no significant changes in the incidence of viral infections during the trial. All participants who returned their supplement completion forms and/or empty supplement containers (9/12) adhered to the supplement schedule, with all achieving the minimum >80% intake.

### 3.1. Participants

All twelve participants (all male) were healthy. The average age was 40.5 years. There was no difference in age (*p* = 0.139) between those on Product A or Product B according to a Kolmogorov–Smirnov test. All participants were healthy and did not have conditions that would affect the study results. None of the participants were anaemic (average haemoglobin 154 g/L; haematocrit 0.46; RBC 5.0 × 106/L).

### 3.2. CD4+/CD8+/CD69+ Analysis

An intention to treat analysis was conducted, as one participant failed to complete all measures. All of this participant’s baseline results were carried forward and used as final results. All participants were used as their own control; this means 24 baseline results were compared against 24 final results (i.e., 12 for Product A and 12 for Product B). All data for lymphocyte counts and percentages of CD3+, CD4+ and CD8+ were normally distributed. There was no difference in the absolute percentages of CD4+ or CD8+ cells after supplementation with bLF, or changes in percentages of CD4+ or CD8+ cells after supplementation (Product A or B).

**Figure 1:**
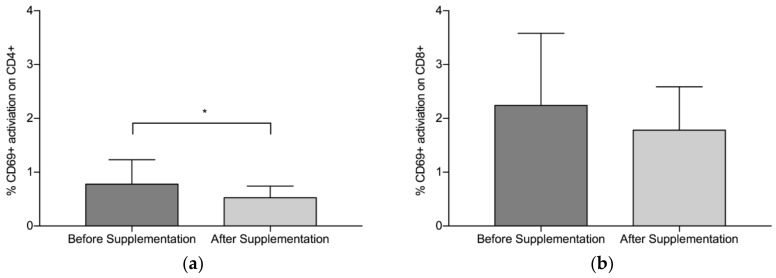
Pooled data from both Product A (*n* = 12) and B (*n* = 12) and both dosages. (**a**) Mean % CD69+ activation of CD4+ before and after supplementation with lactoferrin, (**b**) Mean % CD69+ activation on CD8+ before and after lactoferrin supplementation. Bars indicate standard deviation. * *p* < 0.05.

**Figure 2:**
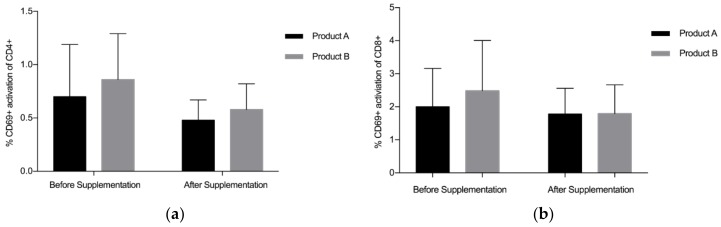
Pooled data from both dosages. (**a**) % CD69+ activation of CD4+ for Products A and B, (**b**) % CD69+ activation of CD8+ for Products A and B. Bars indicate standard deviation. * *p* < 0.05.

**Figure 3:**
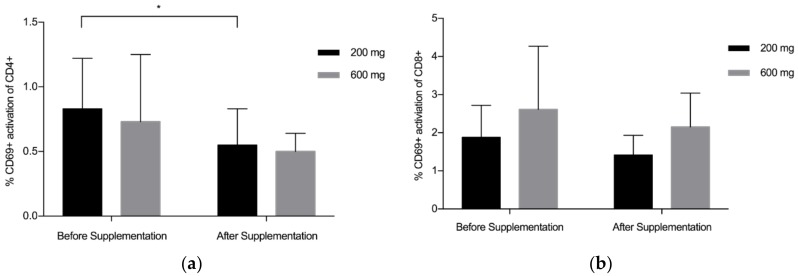
Pooled data from both Product A and B (200 mg, *n* = 12; 600 mg, *n* = 12). (**a**) Mean % CD69+ activation of CD4+ by dose of lactoferrin before and after supplementation; (**b**) Mean % CD69+ activation of CD8+ by dose of lactoferrin before and after supplementation. Bars indicate standard deviation. * *p* < 0.05.

Overall, findings suggest that there is a statistically significant downregulation of CD69+ on CD4+ after supplementation with LF (both Product A and Product B), *p* = 0.008 (Figure 1a). The mean (standard deviation, SD) level of CD69+ activation on the CD4+ was lower after the supplementation period (Before: 0.78 (0.45), After: 0.53 (0.22)). There was a trend towards a statistically significant difference in the activation of CD69+ on the CD8+, *p* = 0.059 (Figure 1b), with lower levels of activation seen after supplementation (Before: 2.24 (1.34), After: 1.78(0.81)). When Product A and Product B were analysed separately, there was a trend towards a difference in the reduction of CD69+ activation on CD4+ with Product A (*p* = 0.075) and Product B (*p* = 0.063), with Product B marginally better (Figure 2a). When Product A and B were analysed separately, there was no significant difference in the reduction of CD69+ activation on CD8+ (Figure 2b). When the different doses were examined separately (but combining both Product A and Product B groups), the 200 mg dose of LF showed a statistically significant reduction in CD69+ activation on CD4+ cells, *p* = 0.006 (Figure 3a), while the reduction in CD69+ activation on CD8+ cells trended toward statistically significant, *p* = 0.058 (Figure 3b). In contrast, there were no statistically significant differences in CD69+ activation on CD4+ cells (*p* = 0.181) or CD8+ cells (*p* = 0.297) for the group taking the higher dose of supplement (600 mg).

### 3.3. Lactoferrin Absorption

#### 3.3.1. Faecal Samples

Levels of both hLF and bLF in faeces were higher post supplementation in the Product B (Inferrin^TM^) group, but lower in the Product A (standard bLF) group, although this did not reach statistical significance. There was a trend toward significance for the increase in bLF in faeces using Product B (*p* = 0.064). When Product B was analysed by dosage, there was an almost significant increase post-supplementation for the 600 mg dose (*p* = 0.055), while there was no significant difference at 200 mg, or for either dose of Product A (Table 2).

#### 3.3.2. Serum Samples

There was no bLF detected in the serum samples tested for any of the participants. The data for all participants was zero. Results for the testing of hLF in serum appeared to have no association with the supplementation with LF in either arm of the study. There was no statistically significant difference between mean baseline and mean final hLF levels at the end of arm 1 (baseline = 2.75 ng/mL; final = 1.29 ng/mL; *p* = 0.055) or arm 2 (baseline = 2.37 ng/mL; final = 2.59 ng/mL; *p* = 0.671) according to a paired *t*-test.

### 3.4. Gut Microbiome

No substantial changes in bacterial populations were detected post-supplementation in the first arm of the trial. This was subjected to statistical analysis in the lab and no statistically significant differences were found. In the second arm of the trial after the two-week washout period, there were some differences before and after supplementation, particularly with the Inferrin^TM^ product (Product B) (see Supplemental Data—Figure S1). It appeared that LF decreased levels of Euryarchaeota, Acidobacteria, Chloroflexi, NC10, and Nitrospirae, and increased levels of Firmicutes and Bacteroidetes (see Supplemental Data—Figure S1).

#### 3.4.1. Metagenomic Sequencing

The top 100 bacterial species in the samples were identified (see Supplemental Data). Not all species could be named as some of the genomes included in the study were yet to be isolated and did not have a formal name. The results of fold changes for each of these bacterial species are graphed for Product A, Product B, and for LF supplementation overall in the Supplemental Data.

## 4. Discussion

This study aimed to evaluate the effect of the microencapsulation of bLF with Progel (Inferrin^TM^) on its absorption, and evaluate the efficacy of bLF (in standard form or as Inferrin^TM^) for improving immune function. It was hypothesised that the extent of absorption of bLF as Inferrin^TM^ would be higher than that of standard bLF not encapsulated with Progel. Twelve healthy male participants undertook at a 10-week double-blind randomised cross-over trial, where each participant was randomly assigned a dose of bLF (200 mg or 600 mg). During the first arm of the trial, participants were randomly assigned to receive the bLF encapsulated in Progel (Inferrin^TM^; Product B) or without encapsulation with Progel (Product A) for 4 weeks. After a 2-week washout participants received the same dose in the other product. 

The main findings from this study are as follows: (1) Inferrin^TM^ at an equivalent bLF dose of 600 mg increased the amount of bLF found in faecal samples, suggesting that Progel microencapsulation protects the bLF from degradation in the digestive system; (2) there is a statistically significant downregulation of CD69+ on CD4+ after supplementation with bLF regardless of whether it was microencapsulated or not; and (3) bLF supplementation as Inferrin^TM^ influences expression levels of bacterial species in the human microbiome, this is potentially attenuated by when consuming Inferrin^TM^.

The functional properties of LF are dependent on the structural conformation of the molecule, any disruption of the structure leads to the loss of functional domains [9]. Receptors for LF are found in the intestinal mucosa and lymphatic tissue of the gut [9]. The gastric and intestinal digestion of LF could affect its functional properties; therefore, conservation of the structural integrity of LF is of importance. Inferrin^TM^ provides a method of preventing the loss of structure in the early stages of digestion [10], allowing the LF to retain its potential health benefits. Lactoferrin absorption was measured by the amount of hLF and bLF found in serum and faecal samples collected before and after supplementation. Previous in-vitro research has shown that Progel microencapsulation protects the bLF from denaturation in gastric fluids, allowing the bLF to reach the intestines [10]. Faecal content of bLF was minimal after supplementation of either product at 200 mg, suggesting that at this dose, the concentration of bLF in the faecal matter is below the limit of detection of the assay. Supplementation with Inferrin^TM^ containing 600 mg bLF led to higher faecal levels of bLF after supplementation, compared to the standard bLF supplement. This result suggests bLF contained in Inferrin^TM^ is protected from denaturation, potentially increasing its ability to reach the intestinal receptors and provide health benefits. This is a highly novel finding of this work. A larger trial would provide more conclusive evidence on Inferrin^TM^ digestion and absorption.

Various white blood cell counts were tested as part of the analysis. CD3+ cell counts represent the total T-cell (T-lymphocyte) count. This count is made up of both CD4+ and CD8+ cells. CD4+ (helper T cells) and CD8+ (cytotoxic T cells) are key modulators of the immune system. CD4+ (helper T cells) send signals to activate the body’s immune response when exposed to a viral or bacterial stimulus [29,30]. CD4+ cells also stimulate macrophages to produce both pro-inflammatory, and anti-inflammatory cytokines. CD8+ or cytotoxic T cells kill infectious targets by inducing apoptosis, or cell death [30]. When disease, inflammation or infection is present, CD69+ activation increases on CD4+ and CD8+ cells, and is used as an early indicator of T-cell activation [31].

The mean level of CD69+ activation on the CD4+ was lower after the supplementation period, suggesting participants’ immune systems were less ‘activated’, potentially due to bLF supplementation. When Product A and Product B were analysed separately, there was a trend towards a difference in the reduction of CD69+ activation on CD4+ with both products, with Product B marginally better. On testing in larger samples (i.e., >12 participants per group), these findings may become statistically significant. Overall, this study suggests bLF supplementation decreases the activation of CD4+ cells by CD69+, potentially indicating participants were less affected by infectious agents, due to bLF supplementation. A previous study supplementing eight healthy males with bLF (100–200 mg) for 14 days showed a significant increase in both CD4+ and CD8+ activation by CD69+ after 16–21 days [26]. The difference in results could be explained by the length of follow up, as our study monitored changes in the immune system during supplementation, not after. A longer trial with a larger sample would provide more clarity around the potential effects of bLF on T cell activation.

It appeared that bLF decreased levels of Euryarchaeota, Acidobacteria, Chloroflexi, NC10, and Nitrospirae, and increased levels of Firmicutes and Bacteroidetes. This was significant for some species when supplemented with 600 mg bLF, regardless of encapsulation. A brief interpretation of these findings is provided below and generally suggests, in concordance with the results for the immune cells, that the bLF supplement had a small effect on immunity and infection. Euryarchaeota are one of the least diverse bacterial species in humans. There are only three species known to be regularly found in humans, and these are referred to as methanogens, or methane-producing bacteria [32]. They are also commonly found at the site of infection, including infected periodontal pockets and tooth root canals [32]. Interestingly they were not identified in the samples in trial arm 1, suggesting they may not be regular colonisers; however, the finding that they were reduced after supplement consumption is worthy of further investigation given their purported associated with infection [33]. Acidobacteria are a more recently discovered phylum and little is known about their function in humans; however, they are a widely distributed bacteria in nature, particularly in soils [33]. They may be associated with carbohydrate, nitrogen and iron metabolism to name a few potential functions [33]. Chloroflexi’s role in human health is not definitive; however, it has been associated with oral infections, with a much higher prevalence (80–90%) in people with periodontitis than those without periodontitis (<10%) [34]. The NC10 phylum was first located in Australia’s Nullarbor Caves [35]. NC10 bacteria are reported as being able to convert methane to carbon dioxide using nitrite [36]. Nitrospirae is a type of bacteria involved in the nitrogen cycle, converting nitrites to nitrates through oxidation [37]. The role of NC10 and Nitrospirae in the human microbiome is unknown. Firmicutes and Bacteroidetes are some of the most abundant bacteria in the human microbiome. Firmicutes is thought to be associated with dietary energy intake and obesity, with those with greater levels of obesity having higher amounts of Firmicutes [38]. It is also associated, in zebrafish, with an increase in number and size of lipid droplets, which promotes fatty acid absorption [39]. The amount of Firmicutes appears to increase more after a meal, while the amount of Bacteroidetes appears to be higher during fasting [40,41]. Therefore, any variations in this group throughout the study cannot be attributed to the supplement, but more associated with variations in dietary intake.

The potential role of recombinant hLF and bLF in immune function and the microbiome has been previously reported. It has been suggested that LF may play an important role in the development and composition of gut microbiota in neonates [42]. In vitro research has shown hLF and bLF significantly promote intestinal proliferation and development through influencing the expression of several pathways, including Ras homolog gene family, member A, Wingless-related integration site/b-catenin, and mitogen-activated protein kinases/extracellular signal-related kinases [27]. A recent review into the role of LF in gastrointestinal development and immune function concluded that LF influenced both systemic and gastrointestinal immune development by providing a balanced T helper cell immune response, and led to higher secretion of anti-inflammatory cytokines in the unstimulated state [43]. It is possible that LF is conferring beneficial effects on the microbiome, which in turn are preventing or reducing the infection rates, and lowering expression of immune cells. Further studies with larger samples are needed to confirm this effect.

There are some limitations to this study. The small sample size is likely under powering analysis, and potentially missing significant results in both the immune cell and absorption analysis. One of the samples from the second arm for the microbiome analysis had low reads and could not be processed and amplified. Fresh, preserved faecal samples were analysed using 16S sequencing. The 16S method extracts DNA from the sample and sequences a small portion of a single bacterial gene (the 16S ribosomal RNA gene). This is used as a ’fingerprint’ to identify which bacterial species are present in the sample. Although the method is more comprehensive than culture-based or PCR-based assays, it does not provide a high resolution of a specific species/strain and is not medically diagnostic. However, this test is economical to run and provides a broad overview of the bacteria, and any changes, in the sample. To manage the limitations of 16S sequencing, metagenomics sequencing was utilised.

## 5. Conclusions

This study aimed to evaluate the effect and efficacy Inferrin^TM^ (bLF with Progel microencapsulation), as seen by its absorption and changes in immune cell biomarkers in healthy male participants. Absorption of bLF appears to be improved with Inferrin^TM^. Additionally, it appears there are some immune modulating effects of bLF on CD69+ activation of CD4+ and potentially CD8+, and some variation in the microbiome post supplementation. This suggests a less activated immune system, potentially due to the positive health benefits of bLF supplementation, although larger studies are required to confirm this effect. 

## Figures and Tables

**Table 1:** Regimen and dosages of assigned products. Product A: Standard lactoferrin; Product B: Inferrin^TM^.

Regime	*n*	Arm 1 (4 Weeks)		Arm 2 (4 Weeks)
1	3	200 mg Product B	Washout (2 weeks)	200 mg Product A
2	3	200 mg Product A		200 mg Product B
3	3	600 mg Product B		600 mg Product A
4	3	600 mg Product A		600 mg Product B

**Table 2:** Bovine faecal lactoferrin content (µg/g) pre and post supplementation dependent on Product and dose, and significance between time points. * *p* < 0.05.

Sample	Pre-Supplementation	Post-Supplementation	*p*-Value
Product A 200 mg	0.53 (1.18)	0.03 (0.06)	0.373
Product A 600 mg	0.04 (0.10)	0.03 (0.05)	0.771
Product B 200 mg	0.08 (0.15)	0.08 (0.18)	0.654
Product B 600 mg	0.11 (0.27)	2.45 (2.41)	0.055

## Supplementary Materials

The following are available online at http://www.mdpi.com/2072-6643/10/8/1115/s1, Table S1: The top 100 bacterial species identified in faecal samples. Figure S1: Phylum-level breakdown of microbial community profiles expressed as relative abundance. The key code indicates whether the sample was pre or post supplementation. Figure S2: Fold changes in top 100 bacterial species for (a) Product A dose 200 mg, and (b) Product A 600 mg. Figure S3: Fold changes in top 100 bacterial species for (a) Product B dose 200 mg, and (b) Product B 600 mg). Figure S4: Fold changes in the top 100 bacterial species. Pooled data for Products A and B.
