# Supplementary material for: Bioavailability of a Novel Form of Microencapsulated Bovine Lactoferrin and Its Effect on Inflammatory Markers and the Gut Microbiome: A Pilot Study
Source: Nutrients. 2018 Aug 17;10(8):1115. doi: 10.3390/nu10081115 (PMC6115941; doi:10.3390/nu10081115)
Supplement: Supplementary file 1 [file nutrients-10-01115-s001.pdf]

Table S1. The top 100 bacterial species identified in Fecal samples by metagenomic sequencing.

Rank	Bacterial species identified
1	Bacteroides vulgatis
2	Prevovtella copri
3	Bacteroides uniformis
4	Novel species
5	Bacteroides stercoris
6	Escherichia coli
7	Faecalibacterium prausnitzii_A
8	Novel species
9	Agathobacter rectalis
10	Blautia wexlerae
11	Novel species
12	Bacteroides ovatus
13	Faecalibacterium prausnitzii_C
14	Novel species
15	Novel species
16	Faecalibacterium prausnitzii_B
17	Novel species
18	Alistipes putredinis
19	CAG37 sp1
20	Novel species
21	CAG94 sp1
22	Parabacteroides distasonis
23	Novel species
24	Roseburia intestinalis
25	Subdoligranulum sp2
26	Novel species
27	Roseburia inulinivorans
28	Collinsella aerofaciens
29	Novel species
30	Ruminococcus_E bromii
31	Anaerostipes hadrus
32	UBA11524 sp1
33	Ruminococcus_B faecis
34	CAG24 sp1
35	Blautia sp2
36	Novel species
37	Oscillibacter sp4
38	Novel species
39	CAG180 sp2
40	Agathobacter faecis
41	Novel species
42	Novel species
43	Bacteroides intestinalis
44	Novel species
45	Eubacterium_K siraeum
46	Novel species

47	Lachnospira eligens
48	Novel species
49	Novel species
50	Bacteroides fragilis
51	Dialister sp1
52	Bacteroides caccae
53	Novel species
54	Novel species
55	Barnesiella intestinihominis
56	Akkermansia muciniphila
57	Bifidobacterium longum
58	Ruminococcus_D bicirculans
59	Novel species
60	CAG349 sp1
61	Novel species
62	CAG217 sp1
63	Sutterella
64	Alistipes shahii
65	Novel species
66	Novel species
67	Novel species
68	Novel species
69	Bifidobacterium adolescentis
70	Novel species
71	F23-B02 sp3
72	Phascolarctobacterium
73	Novel species
74	Oscillibacter sp6
75	Novel species
76	Ruminococcus_E sp1
77	Novel species
78	Novel species
79	Novel species
80	Bacteroides finegoldii
81	PeH17 sp1
82	Novel species
83	Novel species
84	Novel species
85	Novel species
86	CAG177 sp1
87	Ruminococcus_B lactaris
88	Novel species
89	Catenibacterium mitsuokai
90	CAG279 sp1
91	Novel species
92	Novel species
93	Novel species
94	Novel species
95	Novel species

96	Roseburia hominis
97	CAG510 sp1
98	Bilophila wadsworthia
99	Novel species
100	Prevotella sp13

Figure S1: Phylum – level breakdown of microbial community profiles expressed as relative abundance. The key code indicates whether the sample was pre or post supplementation.

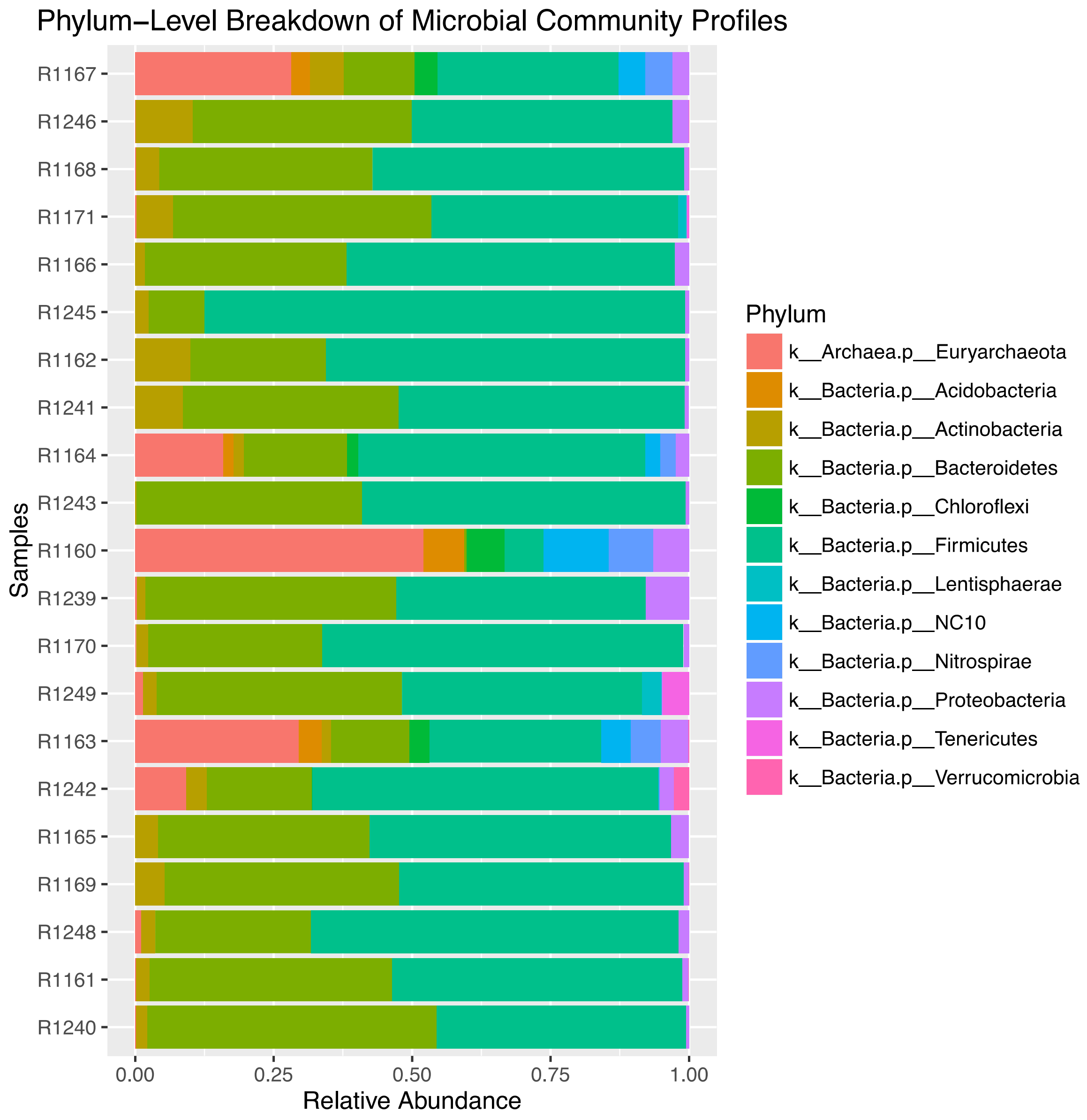

Before	After
R1160	R1239
R1161	R1240
R1162	R1241
R1163	R1242
R1164	R1243
R1165	dropped out

R1166	R1245
R1167	R1246
R1168	Low reads
R1169	R1248
R1170	R1249
R1171	R1250

a)

b)

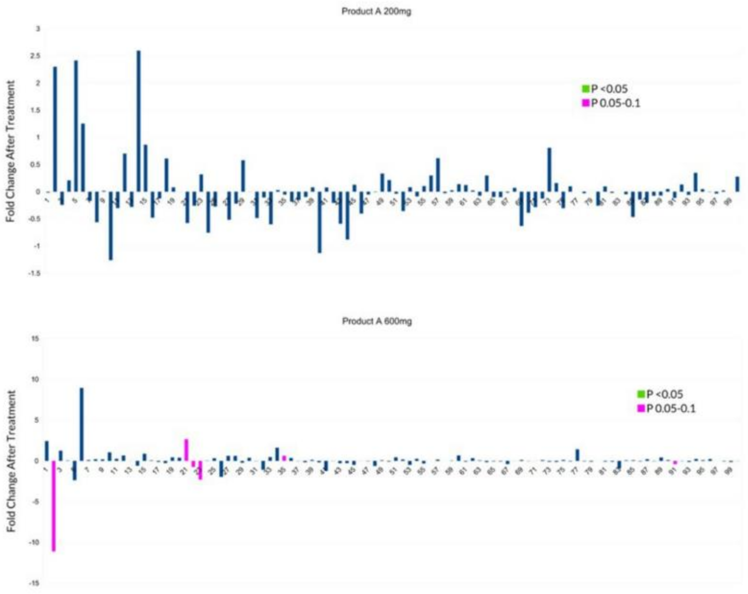

Figure S2~~1~~. Fold changes in top 100 bacterial species for a) Product A dose 200mg, and b) Product A 600mg.

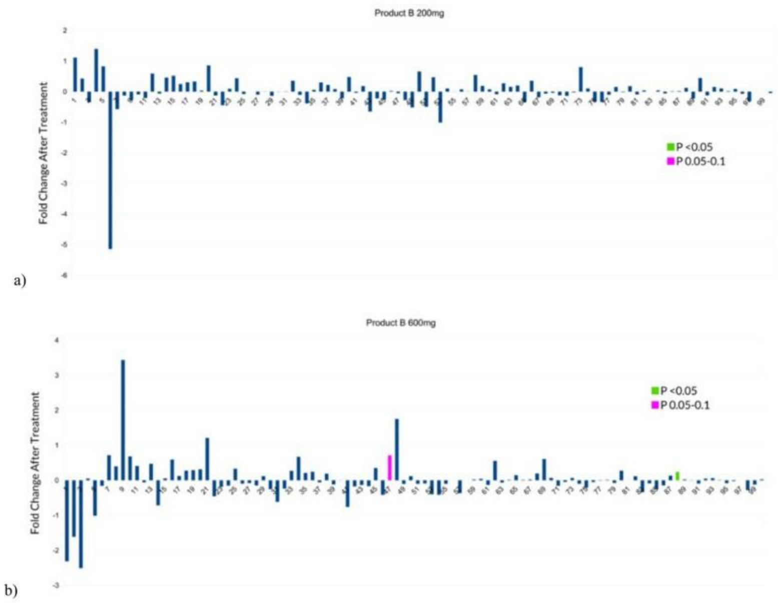

Figure S32. Fold changes in top 100 bacterial species for a) Product B dose 200mg, and b) Product B 600mg).

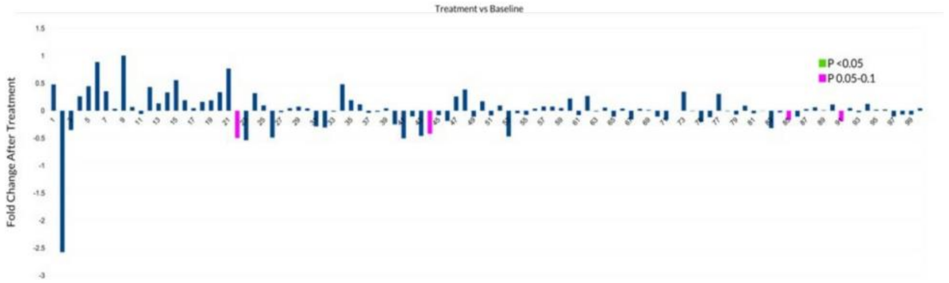

Figure S43. Fold changes in the top 100 bacterial species. Pooled data for both products.
